# Using Random Forest to Improve the Downscaling of Global Livestock Census Data

**DOI:** 10.1371/journal.pone.0150424

**Published:** 2016-03-15

**Authors:** Gaëlle Nicolas, Timothy P. Robinson, G. R. William Wint, Giulia Conchedda, Giuseppina Cinardi, Marius Gilbert

**Affiliations:** 1 Biological Control and Spatial Ecology, Université Libre de Bruxelles, Brussels, Belgium; 2 Fonds National de la Recherche Scientifique, Brussels, Belgium; 3 International Livestock Research Institute (ILRI), Livestock Systems and Environment (LSE), Nairobi, Kenya; 4 Environmental Research Group Oxford (ERGO) - Department of Zoology, University of Oxford, Oxford, United Kingdom; 5 Animal Production and Health Division (AGA), Food and Agriculture Organization of the United Nations (FAO), Rome, Italy; DOE Pacific Northwest National Laboratory, UNITED STATES

## Abstract

Large scale, high-resolution global data on farm animal distributions are essential for spatially explicit assessments of the epidemiological, environmental and socio-economic impacts of the livestock sector. This has been the major motivation behind the development of the Gridded Livestock of the World (GLW) database, which has been extensively used since its first publication in 2007. The database relies on a downscaling methodology whereby census counts of animals in sub-national administrative units are redistributed at the level of grid cells as a function of a series of spatial covariates. The recent upgrade of GLW1 to GLW2 involved automating the processing, improvement of input data, and downscaling at a spatial resolution of 1 km per cell (5 km per cell in the earlier version). The underlying statistical methodology, however, remained unchanged. In this paper, we evaluate new methods to downscale census data with a higher accuracy and increased processing efficiency. Two main factors were evaluated, based on sample census datasets of cattle in Africa and chickens in Asia. First, we implemented and evaluated Random Forest models (RF) instead of stratified regressions. Second, we investigated whether models that predicted the number of animals per rural person (per capita) could provide better downscaled estimates than the previous approach that predicted absolute densities (animals per km^2^). RF models consistently provided better predictions than the stratified regressions for both continents and species. The benefit of per capita over absolute density models varied according to the species and continent. In addition, different technical options were evaluated to reduce the processing time while maintaining their predictive power. Future GLW runs (GLW 3.0) will apply the new RF methodology with optimized modelling options. The potential benefit of per capita models will need to be further investigated with a better distinction between rural and agricultural populations.

## Introduction

Current and spatially detailed assessments of the various impacts and benefits of livestock production in terms of human and animal health, environment and livelihoods rely heavily on reliable high-resolution data on the distribution of farm animals. The distribution of animal hosts is also key to veterinary epidemiology as it provides the denominator for any prevalence measure, and provides a framework for prioritising surveillance, prevention and control of livestock diseases. In many countries, livestock census data are available, but their quality, resolution and timeliness are highly variable and require harmonization and standardization. The Gridded Livestock of the World (GLW) project initiated by Food and Agriculture Organization (FAO) in the early 2000s aimed to address these needs by providing estimates of livestock and poultry numbers as raster Geographical Information System (GIS) layer, where a measure of density is provided for each square pixel, for example with a resolution of 10 km, instead of one measure by administrative unit. The initiative involved the compilation and geo-referencing of sub-national livestock survey and census data from diverse sources, and the downscaling of these numbers to the pixel level based on a set of spatial covariates available at high spatial resolution and with global coverage [1]. Recent examples of uses of these data are a global assessment of antimicrobial usage in food animals [2], spatial predictions of avian influenza H7N9 risk in Asia [3], revised assessments of green house gas emissions due to livestock [4,5], and estimates of poor livestock keepers potentially affected by zoonotic diseases [6]. The importance of accurate, detailed and timely estimates of global livestock distributions is further enhanced by the rapid changes in transition economies, where intensification of livestock production is changing their distribution in space and time [7], with substantial potential human and animal health implications [8,9].

In 2007, the first version of the GLW database (GLW 1.0) was produced [10] and disseminated through the FAO Geonetwork spatial data repository (http://livestock.geo-wiki.org/) at a spatial resolution of 0.05 decimal degrees (about 5×5 km at the equator) based on census data of the late 90's and early 2000’s. This first version of GLW included distribution maps of cattle, buffalos, sheep, goats, pigs and poultry (chicken, ducks and geese pooled together), with modelled country totals adjusted to match the official FAOSTAT national estimates for the reference year 2005. The statistical method employed to downscale census data consisted of linear multiple regressions stratified by zones sharing similar eco-climatic conditions [10], and the processing was labour intensive. In recent years, the methodology has been standardized and automated as a series of scripts performing the sequential processing steps. These were initially evaluated for poultry data in Asia [11,12]. In parallel, other authors evaluated different downscaling techniques and confirmed the value of regression-based methods over simpler land-use based models [12,13]. At the same time, contemporary livestock data were being collected by FAO for more species with much higher levels of spatial detail, and higher resolution spatial covariates were assembled. The integration of these new data sets with streamlined processing and improved spatial predictors lead to the publication and dissemination of the second version of the GLW database (GLW2) in 2014, which included 30 arc-second (c. 1 km) resolution distribution data for cattle, pigs, chickens, ducks, and later, sheep and goats [14]. The GLW2 processing also included a bootstrapping procedure to assess internal variability of the predictions, though the underlying methodology remained similar to that used to develop GLW 1.0, *i*.*e*. linear multiple regressions stratified by eco-climatic zones or livestock production systems. Though these represented significant methodological advances producing the GLW2 maps was extremely computationally-intensive, posing a significant constraint to providing regular updates as new census data became available.

In recent years, machine-learning techniques such as Boosted Regression Trees (BRT), Random Forest (RF), Neural Networks or Maxent have been shown to yield better predictions than linear modelling methods in species distribution modelling [15–17]. RF has also been shown to give better predictions than previous methods in downscaling human population census data to pixel level [18,19], which is very similar in its objectives to downscaling livestock census and survey data. The main objective of this study was therefore to test and evaluate RF models in comparison to the stratified regressions used for GLW2.

Livestock distributions are strongly related to those of stockholders. So, in contrast to wildlife species, farm animals cannot be found in remote areas where no people live. Even in the rare circumstances where they are raised as freely grazing herds the animals generally remain close to settlements especially when considered at the regional or country scale. Even nomadic livestock production systems in sub-Saharan Africa are not totally independent from settlements or locations with water access where populations are present.

A potential drawback of the GLW2 methodology, where livestock densities are expressed and modelled per unit of land, is that nothing prevents the model from predicting non-zero livestock numbers in areas where people are absent. An alternative approach, that we evaluated here, was to model animals per capita, based on recently available high resolution human population data [20–22], with a view to intrinsically constraining livestock predictions to areas where there is human activity.

Finally, some of the modelling options in GLW2, such as the spatial resolution of the modelling, the number of bootstraps and the selection of predictor variables, had been chosen rather arbitrarily. The comprehensive assessment undertaken here offered the opportunity to carefully examine some of these, with the aim of simplifying the procedure whilst maintaining the predictive capacity.

In summary this study examines whether the GLW2 downscaling methodology could be improved by i) the use of RF models, ii) a change in the dependent variable from livestock number per unit of land to numbers per capita, and iii) adjusting some of the modelling options.

## Materials and Methods

### GLW 2 methodology

The GLW2 methodology is fully described in Robinson et al. [14] and is only briefly summarized here, with more emphasis on the parts of the processing that we aimed to improve. The methodology relies on several sequential steps:

1. the density of animals per km^2^ of suitable land is estimated in all polygons corresponding to the sub-national livestock data and transformed to its logarithmic value (base 10);
2. a large set of sample points is built to cover the modelling extent (a minimum number of one point per input polygon with non-zero data, with a minimum density of 30 points per 10,000 km^2^ of land in large polygons) and values for observed densities, predictor variables and stratification (the global livestock production systems (GLPS version 5) [23], biomes stratification [24] and 25 discrete ecological zones (EZ25) [25]) are extracted from their respective polygons (census data) or pixels (predictor variables);
3. the sample file is divided into *n* sub-samples for bootstrapping the analysis;
4. each sub-sample is used to build a series of multiple regressions, with one multiple regression per stratum.
5. the models for each stratum are evaluated, and the best applied to the predictor variables to obtain the single best predicted value for each pixel;
6. the predicted values are averaged over the *n* bootstraps;
7. post-processing is carried out to correct polygon totals (to make sure that the sum of the grid cell values within a polygon is equal to the observed totals) and then by country totals (to make sure that the sum of grid cell values in a country matches the FAOSTAT official total for a specified base year).

The spatial covariates used to make the predictions include Fourier-transformed remotely sensed variables (two vegetation indices, the day and night land surface temperature and the band 3 middle-infra-red) [26], eco-climatic variables (length of growing period and annual precipitation), topographic variables (elevation and slope) and anthropogenic variables (human population density and travel time to major cities). Altogether, the full list of spatial predictor variables amounts to 72, detailed in Table 1.

**Table 1:** Summary of predictor variables.

Type of variable	Predictor variables	Ap	Fp	Source
**Vegetation and climate**	12 Fourier-derived variables from MIR^a^	x	x	[26]
	12 Fourier-derived variables from LST^b^			
	day	x	x	
	night	x		
	12 Fourier-derived variables from			
	NDVI^c^	x	x	
	EVI^d^	x		
	Green-up (annual cycle 1 and 2)	x		[27]
	Senescence (annual cycle 1 and 2)	x		
	Length of Growing Period (LGP)	x	x	[28]
	Precipitation	x	x	[29]
	Cropping intensity	x	x	[30]
	Forest Cover	x	x	[31]
**Topography**	GTOPO30 Elevation	x	x	
	GTOPO30 Slope	x	x	
**Demography**	Human population in 2010^e^	x	x	Worldpop [18]
	Travel time to places with > 50,000 inhabitants	x	x	[32]

^a^ Middle Infra-Red

^b^ Land Surface Temperature

^c^ Normalized Difference Vegetation Index

^d^ Enhanced Vegetation Index

^e^ Country totals adjusted to UN values in 2006 (http://www.un.org/esa/population/)

Ap: all predictors of GLW2; Fp: reduced set of predictor variables

### Training data

The livestock data sets used in this evaluation were cattle in Africa and chickens in Asia; both previously described in Robinson et al. (2014). Cattle in Africa were chosen in order to be representative of comparatively extensive ruminant farming, strongly linked to the land resource for which they are dependent for fodder, whilst still presenting a wide range of cattle farming systems and densities. Chickens in Asia were chosen to represent monogastric species (poultry and pigs), which can be raised at varying degrees of intensity and are less closely associated with specific environmental drivers but probably more with demographic variables. The rationale for considering both of these very different species and continents was to include a wide variety of agro-ecological situations in which to test the proposed methodological improvements, and so provide general rules that would be applicable to other livestock species and regions, though this remains to be confirmed by future work.

### Evaluation procedure

**Fig 1:**
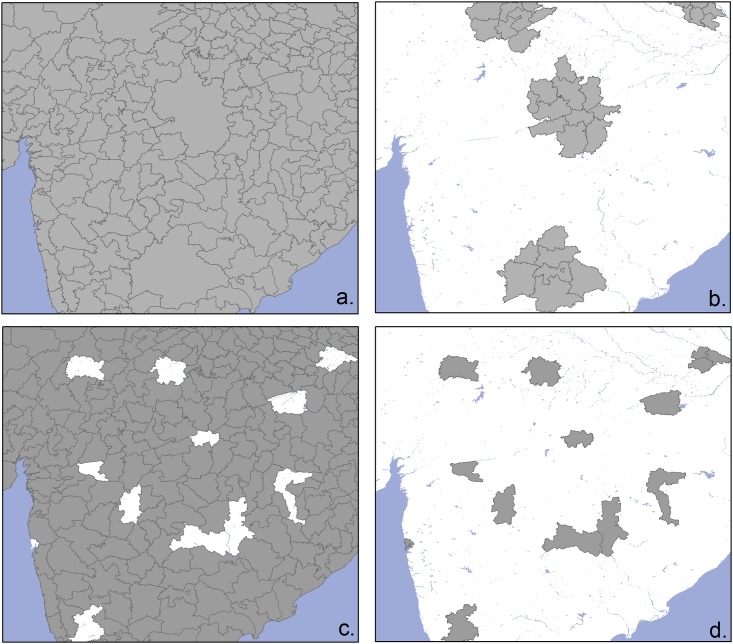
Illustration of training polygons (grey polygons in a and c) and test polygons (grey polygons in b and d) used to evaluate the goodness of fit with the downscaling (a—b) and gap-filling (c—d) methodology.

We used two approaches to evaluate the different methods. The first, termed “downscaling” aimed to quantify the capacity of the algorithm to spatially disaggregate the observed livestock data. We artificially degraded the spatial resolution of the input data by randomly distributing circles with three different radii (100 km, 200 km and 500 km) over the two regions, summing the values of animal census count of all administrative unit polygons falling within each circle. These radii were chosen by analogy with a situation where only one animal value would be available for a spatial unit with the size of an entire province or country. This provided a degraded data set, with many large circles each with a single average value of livestock density, instead of different values for several smaller polygons. This degraded data set was used to train the model, and the evaluation was carried out by comparing the values predicted by the model with those of the original detailed polygons located within the circles. The second approach, termed “gap-filling” simply aimed to evaluate the capacity of the model to predict values in areas with no census data. A set containing 30% of the administrative unit polygons with known animal densities was taken out from the training set, and the values predicted by the model were compared to the observed densities. Fig 1 presents an illustration of a typical training and evaluation set for both approaches in India. Since both approaches involved the random distribution of polygons for model evaluation, they were repeated to ensure that the result would not be influenced by a particular random sample. The random distribution of circles and the merging of polygons was repeated three times for each circle size, resulting in 3 x 3 runs for each combination of species and continent. The simpler gap-filling procedure, requiring the random selection of polygons to be excluded from the processing, was repeated 25 times. Two metrics were used to quantify the goodness of fit between observed and predicted densities: the correlation coefficient (COR) and the root mean square error (RMSE). A correlation coefficient provides an indication of precision, i.e. how closely the observed and predicted values agree in relative terms, with a perfect correlation equal to one [33]. RMSE provides an estimate of accuracy based on the discrepancy between the observed and predicted values [33]. COR and RMSE for both downscaling and gap-filling were estimated for different polygon-size bins, so as to be able to measure the accuracy associated with predictions aggregated at different polygon sizes. So, the full evaluation of each method involved 18 runs (9 for gap-filling and 9 for downscaling) for each species leading to a total of 36 different runs.

### Evaluated factors

First, we compared stratified regression (used in GLW 2) against RF as the statistical modelling method. Statistical approaches start the model fitting by assuming an appropriate data model, and related model parameters are then estimated from the data. By contrast, RF is a machine learning technique, which avoids starting with a data model but rather uses an algorithm to learn the relationship between the response and its predictors. RF combines the prediction of a high number of classification trees in an ensemble, non-parametric approach [17]. The RF algorithm for regression works by: i) drawing *n* bootstrap sub-samples from the original data; ii) growing unpruned regression trees by randomly sampling *m* variables out of the list of predictors and choosing the best split from those predictor variables for each of the bootstrap samples (i.e. each tree) and iii) generating a final predicted value by averaging the prediction of the *n* trees. Compared to other methods, RF is able to model complex interactions among predictors [34] and was recently shown to provide highly accurate results when modelling human population densities [19]. Another reason for choosing RF over other machine learning techniques, such as BRT for example, is that RF predictions are less sensitive to variations in the selected initial parameters. The RF models were implemented with the following arbitrarily set parameters: i) a third of the number of variables were randomly selected for building each tree; ii) the number of trees was estimated as the number of sample points divided by 20, with a minimum of 100; iii) the node size was estimated as 1/1,000^th^ of the number of sampling points, with a minimum node size of 5. Explanatory tests indicated that the predictive performances had a fairly low sensitivity to these parameters. The entire processing was carried out using R 3.2.1, using the randomForest package 4.6–10 [35] for building the RF models and the doParrallel package of parallelisation [36].

Secondly, we evaluated the predictive accuracy of both SR and RF models when the dependent variable was the number of animals per capita (PC) rather than absolute densities (DN). In each input polygon, we divided the animal count by the summed rural population to get the observed number of animals per capita. At the end of the modelling process, we back-transformed predicted per capita pixel values into densities by multiplying them by the rural population and dividing the result by the pixel area. As input data for rural population, we simply applied the Global Land Cover GLC2000 urban mask [37] over the human population WorldPop database of Africa and Asia [18,20] so as to exclude urban populations.

Thirdly, we evaluated the impact of spatial modelling resolution by running models at two spatial resolutions: 0.0083333 and 0.083333 decimal degrees per pixel, which were termed 1k or 10k models, respectively. These two resolutions are typically used for country-scale and global-scale analyses, respectively.

The fourth comparison made related to the number of predictor variables used. The initial GLW2 procedure used 72 predictor variables as potential inputs for the model. We investigated whether a reduced set of predictor variables could perform equally well for a given modelling method. The set with all predictor variables (AP) included all those in the original GLW2 procedure, as well as cropping intensity and forest cover [27] [28]. The set with fewer predictor variables (FP) excluded the Fourier-transformed night Land Surface Temperature (12 variables), Enhanced Vegetation Index (12 variables) and the vegetation phenology variables (4 variables). These were removed as being potentially redundant with Fourrier-transformed day Land Surface Temperature (12 variables), Normalized Difference vegetation Index (12 variables) and length of growing period (4 variables). The reduced list included 36 spatial predictor variables.

Finally, to explore the effect of the number of bootstraps on the prediction accuracy we simply assessed the goodness of fit of the predictions made with 1, 5, 10, 15, 20 and 25 bootstraps using the standard GLW2 procedure. All other comparisons of modelling options (SR vs RF, DN vs PC, etc…) were then made with a constant, but reduced number of bootstraps (see results) with a training set comprising 70% of the input polygons being randomly selected from the full data set; the other 30% being used for evaluation.

Training data and factors tested in the different evaluations are summarized in Table 2.

**Table 2:** Training data and factors tested in the different evaluations.

**Training data**	**n**	**Description**
Training dataset	9	3 radius sizes, 3 replicates for each
Continent/species	2	cattle in Africa, chickens in Asia
**Factor**	**n**	**Description**
Modelling technique	2	Stratified Regression (SR) / Random Forest (RF)
Dependent variable	2	Suitability corrected livestock density (DN) / Animals per rural person (PC)
Resolution	2	0.0083333 (1 km) / 0.083333 (10 km) dec. degrees
Predictors	2	Full set (AP) / Reduced set (FP)
Number of bootstraps	6	1, 5, 10, 15, 20 or 25 bootstraps

## Results

Throughout the analysis of our results, we found extremely similar results between the goodness of fit metrics provided by COR and RMSE. Runs with highest COR corresponded to low RMSE values and vice-versa. We therefore chose to illustrate the results only using the COR between observed and predicted densities. In addition, although there was somewhat more variation, the results of the downscaling and gap-filling comparisons were also similar so we chose only to present the results of downscaling evaluation in the main text of the paper, and consigned the equivalent results for gap-filling assessments to supplementary information (S1 File).

**Fig 2:**
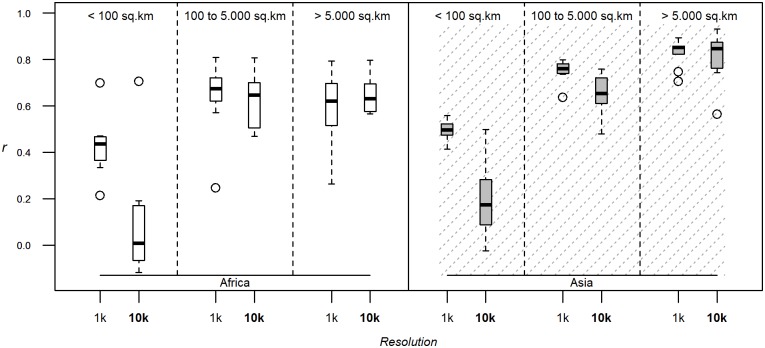
Number of bootstraps. Correlation coefficients between log-transformed observed and predicted densities evaluated through downscaling, using the stratified regression modelling method, density as dependent variable, the full set of predictor variables, the 1 km resolution models, and varying the number of bootstraps. The correlation coefficient is estimated by breaking down evaluation polygons by their size, and for both species and continents.

We first established that the standard GLW2 could be implemented using fewer bootstraps at no significant predictive cost. Fig 2 shows that for all sizes of polygons, and for both Asia and Africa, there was no real gain in predictability beyond 10 bootstraps. Therefore, the effect of all other factors outlined in Table 2 was assessed using 10 bootstraps only.

**Fig 3:**
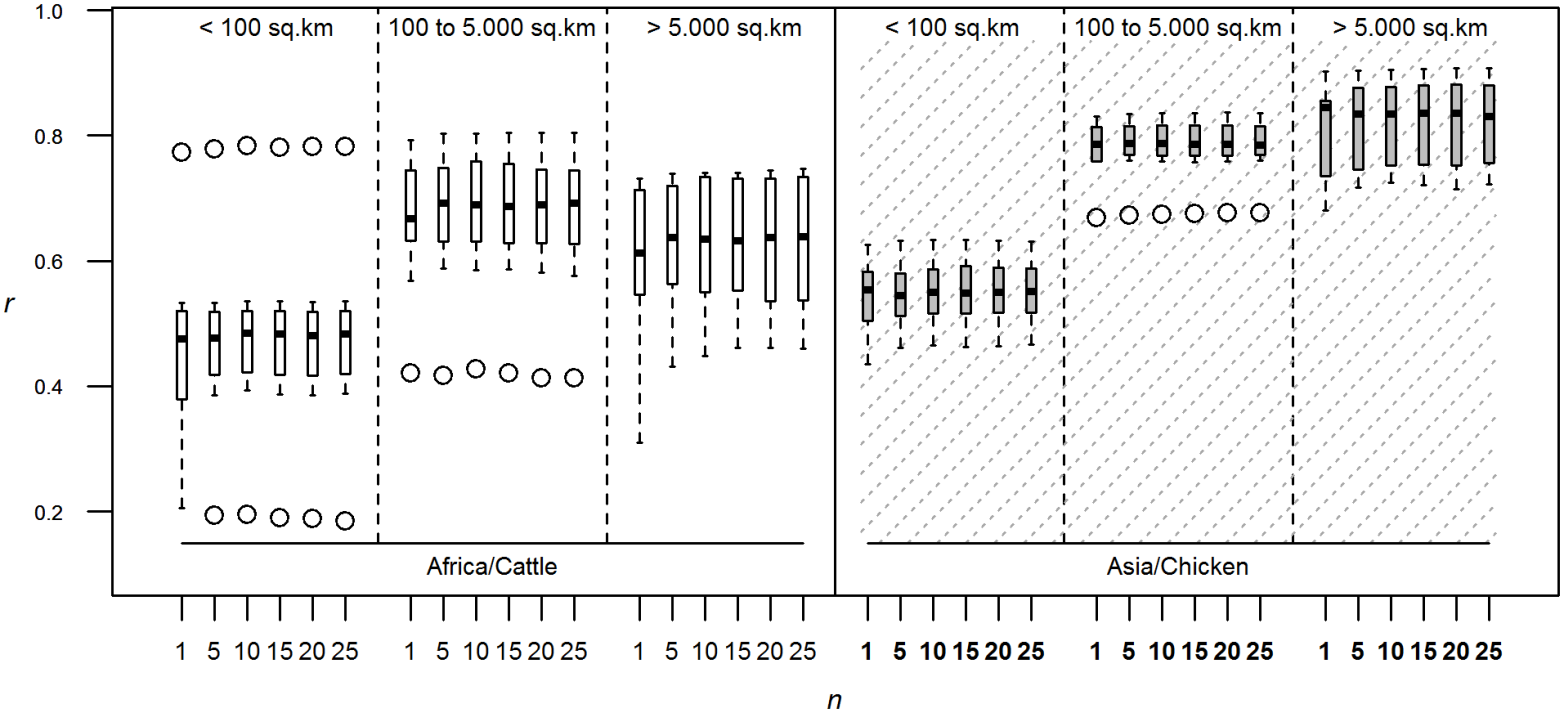
Resolution. Correlation coefficients between log-transformed observed and predicted densities evaluated through downscaling, using the stratified regression modelling method, density as dependent variable, the full set of predictor variables, 10 bootstraps, and varying the spatial resolution of the modelling process (1 km: 0.0083333 decimal degrees resolution; 10 km: 0.083333 decimal degrees resolution). The correlation coefficient is estimated by breaking down evaluation polygons by their size, and for both species and continents.

As a second stage, and using the GLW2 methodology, we compared the goodness of fit of runs carried out at the two spatial resolutions of 1 km and 10 km. As expected, and highlighted in Fig 3 (gap-filling method in Fig A in S1 File), the main benefit of the high resolution modelling was obtained for the smallest polygons, with sizes lower than 100 km^2^. More surprisingly, the largest polygon values appeared to be slightly better predicted by the 10 km models. However, since the primary objective of the GLW algorithm is to downscale the available census data, the benefit of high resolution modelling was considered more important. The following sequence of statistics were therefore estimated based on the high resolution models.

**Fig 4:**
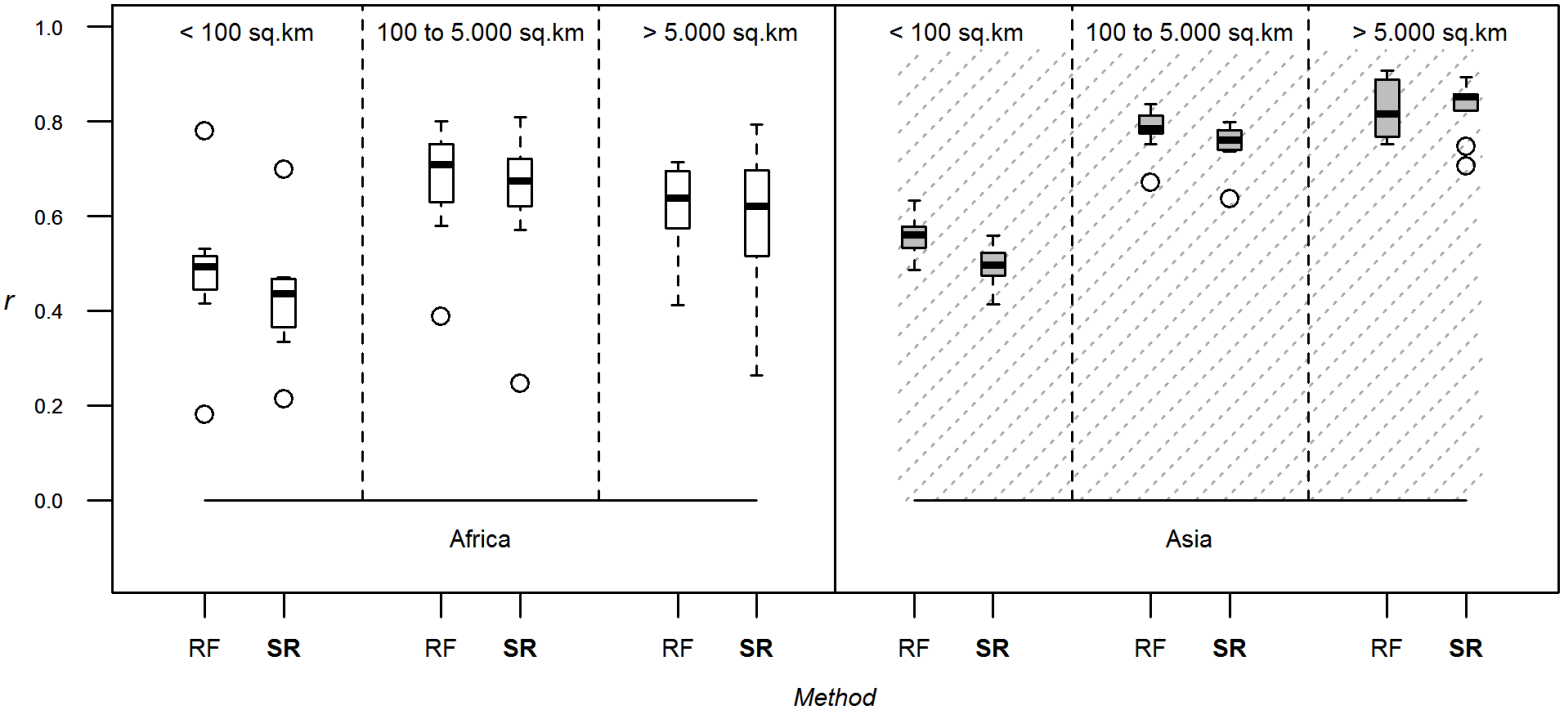
Methods. Correlation coefficients between log-transformed observed and predicted densities evaluated through downscaling, using density as dependent variable, the full set of predictor variables, 10 bootstraps, 1 km resolution modelling and varying the modelling method (SR: Stratified regression corresponding to GLW2; RF: Random Forest). The correlation coefficient is estimated by breaking down evaluation polygons by their size, and for both species and continents.

A considerable improvement in predictive power was observed when RF was used instead of SR (Fig 4) and the gains were more evident for gap-filling than downscaling (Fig B in S1 File).

**Fig 5:**
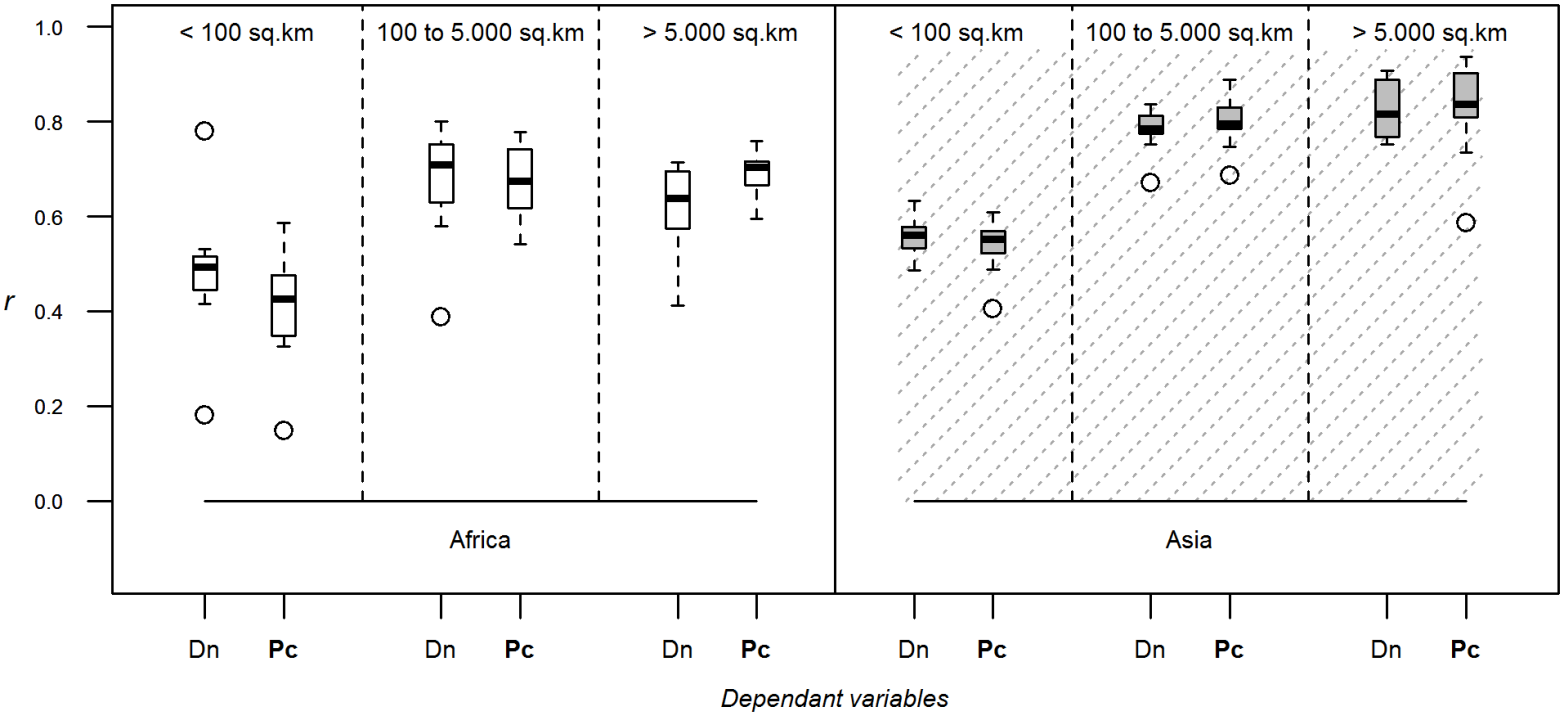
Dependant variable. Correlation coefficients between log-transformed observed and predicted densities evaluated through downscaling, using Random Forest as modelling method, the full set of predictor variables, 10 bootstraps, 1 km resolution modelling and varying the dependent variable (Dn: suitability-corrected density corresponding to GLW2; Pc: number of animals per capita). The correlation coefficient is estimated by breaking down evaluation polygons by their size, and for both species and continents.

In comparing predictions based on the number of animals per capita against those per unit of land area there was no consistent improvement in predictive power (Fig 5) and different results were obtained for the two species and regions and for downscaling compared to gap-filling (Fig C in S1 File). There was a marked increase in accuracy for cattle in Africa and for the smallest polygon size class, but this result was not apparent for the other size classes, nor for chickens in Asia.

**Fig 6:**
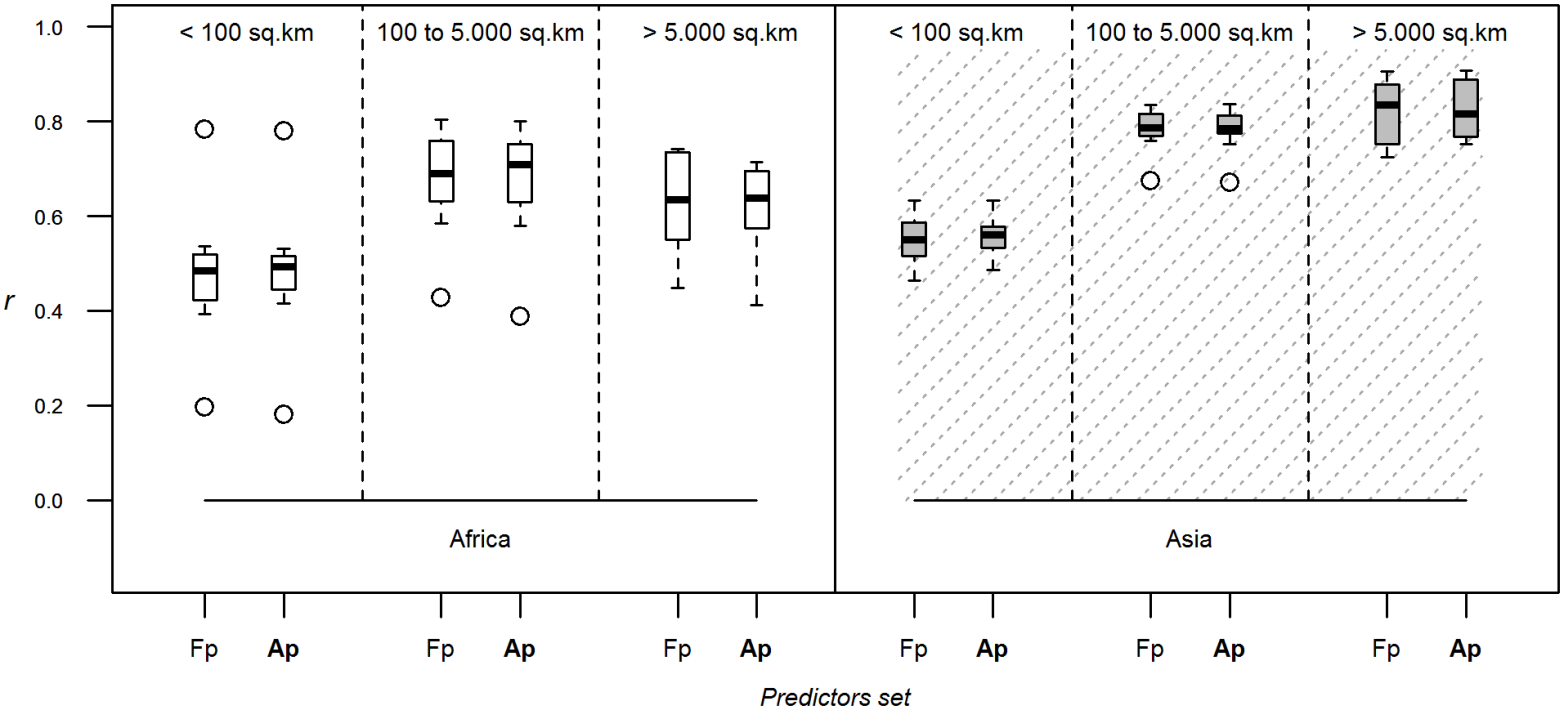
List of predictors. Correlation coefficients between log-transformed observed and predicted densities evaluated through downscaling, using Random Forest as modelling method, density as dependent variable, 10 bootstraps, 1 km resolution modelling and varying the set of predictor variables variable (Ap: all predictors corresponding to GLW2; Fp: reduced set of predictor variables). The correlation coefficient is estimated by breaking down evaluation polygons by their size, and for both species and continents.

Finally, using a reduced set of predictor variables did not lead to any important reduction in predictive power (Fig 6 and Fig D in S1 File).

**Fig 7:**
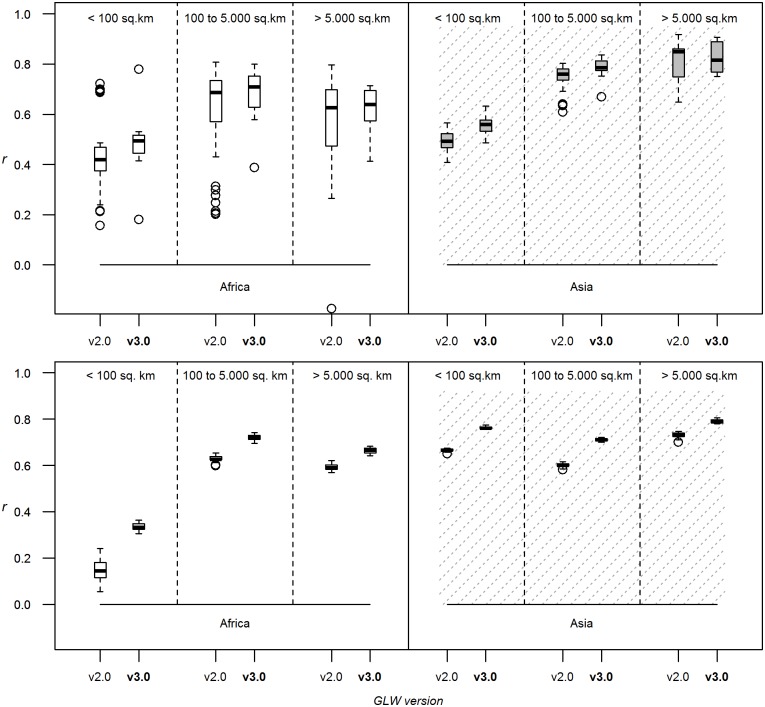
GLW2 vs GLW3. Correlation coefficients between log-transformed observed and predicted densities evaluated through downscaling (top) and gap-filling (bottom) for both the GLW2 methodology (stratified regression modelling, density as dependent variable, 25 bootstraps, 1 km resolution modelling, full set of predictor variable) and the proposed GLW3 methodology (random forest modelling, density as dependent variable, 10 bootstraps, 1 km resolution modelling, reducted set of predictor variables). The correlation coefficient is estimated by breaking down evaluation polygons by their size, and for both species and continents.

**Fig 8:**
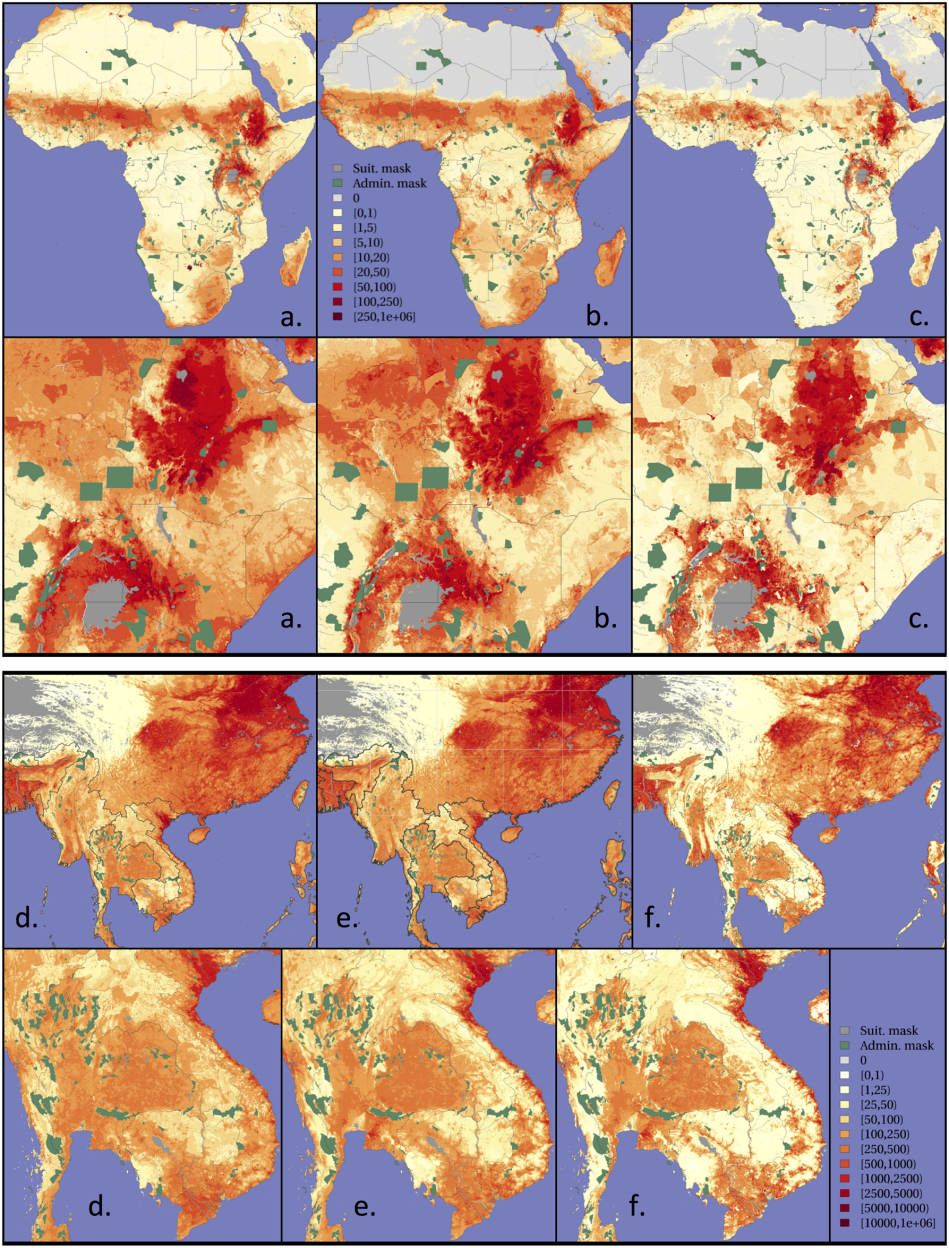
Predicted distribution of cattle in Africa (top) and chickens in Asia (bottom) from the GLW2 methodology (a and d), the proposed GLW3 methodology (b and e) based on random forest, and of GLW3 methodology using animals per capita instead of absolute density (c and f). The data used to produce these maps were all from public sources, and the country limit data are from the FAO Global Administrative Unit Layers (GAUL) database.

Pulling these results together, Figs 7 and 8 illustrate the areas where the GLW2 methodology can be improved, towards a new, third version (GLW3): i) reducing the number of bootstraps to 10, ii) replacing SR with RF, and iii) using a reduced set of predictor variables. Together, these changes result in considerable predictive benefits for the proposed GLW3 methodology (Fig 7). The collective benefits are more apparent when evaluated through gap-filling than through downscaling. Other factors tested here that could have potentially reduced processing time (e.g. modelling a lower spatial resolution) or improved accuracy (e.g. modelling per capita instead of absolute densities) were dismissed for integration into the GLW3 methodology.

Fig 8 shows the mapped outputs of GLW2 and GLW3 for cattle in Africa and chickens in Asia. The mapped output of GLW3 applied to the number of animals per capita is also presented for comparison.

## Discussion

Many methodological choices made in the GLW2 were based on logical assumptions, experience and on published information, but their merits were never systematically evaluated. Several results outlined here simply allowed us to refine some of the initial GLW2 options, such as the number of bootstraps, the spatial resolution of the modelling and the set of predictor variables offered. We found that we were able to produce models with equivalent levels of predictive power using fewer bootstraps (10 instead of 25), we confirmed the expected benefit of high resolution modelling in further downscaling small spatial units, and we were able to reduce the set of predictor variables included in the modelling. The result on the modelling resolution assessments also indicates that runs carried out at 10 km resolution provide perfectly suitable outputs as long as the size of the unit is not too small, i.e. accurate 10 km resolution continental scale outputs could be achieved through modelling at 10 km rather than aggregating 1 km models to 10 km outputs. These results can lead to dramatic reductions in processing time.

We also evaluated two more fundamental changes to the modelling approach. Firstly, RF models clearly outperformed SR, for all size classes of polygons, and regardless of whether the objective was downscaling or gap-filling. This is perhaps the most important result of this study and will result in the most profound change to the GLW methodology since its inception. Machine learning techniques such as RF have been shown to be particularly efficient at predicting spatial patterns in species distribution models compared to more traditional linear models [15]. In downscaling applications, machine learning has been used to process climate [38] and vegetation [39] data. The closest parallel to this study is the work made on improving methods for downscaling human population census made by the Worldpop consortium, who recently found RF models to provide better predictions than previous land-use based downscaling modelling approaches [19]. These findings were corroborated in a separate, unrelated study [16]. We found similar results for both cattle in Africa and chicken in Asia species and continents, and future work should confirm this increased accuracy for other species and at a global scale.

Secondly, made possible by the growing availability of high quality and spatially detailed human population distribution data, we could test the possibility of modelling animals per capita instead of animals per unit of land area. This would have the benefit of intrinsically preventing the model from predicting non-zero densities in unpopulated areas. However, our analyses found no systematic improvements to result from per capita models. Interesting results were observed in Africa using the gap-filling evaluation method, but this was not replicated in the downscaling evaluation, nor apparent for all size classes or for chickens in Asia. However, the spatial pattern of density calculated from per capita models differed (Fig 8) with more contrasted patterns for the per capita model outputs. We therefore feel that this approach has potential and will continue to investigate it. A possible explanation for these variable results is that we currently have a fairly blunt distinction between urban and rural areas, based on the urban class of a global land use map. However, the number of animals per capita is likely to show a more gradual gradient from the most rural areas to the urban centres so the outputs of the current per capita models tend to overestimate animals in the -highly populated peri-urban areas and to underestimate them in the most sparsely populated rural areas. Future attempts to model animals per capita should use more refined definitions of population as denominator. A further caveat to this is that we are using rural population, which does not equate directly to the agricultural population, which is what we really want to get at. There is a number of approaches to estimating agricultural population that we are exploring to this end.

Although not the primary purpose of this study, we observed an overall higher accuracy in predictions of chickens in Asia than of cattle in Africa. This reiterates the findings of Robinson *ET AL*. (2014) and probably relates to the stronger anthropogenic determinants of chicken distribution, as compared to cattle.

The new Version 3 of GLW will therefore include RF as the modelling method, with 10 bootstraps and a reduced set of predictor variables. The evaluations carried out in this study necessitated moving the processing from desktop computers to a cluster where we could take advantage of parallel computing. This, together with changes in the methodology, has reduced the processing time from 7 days to 17 hours for a run over Asia at 1 km resolution. This presents the possibility to implement global runs at 1 km in less than 24 hours for each species that can be updated any time that new subnational data become available.

Several further tests and developments of GLW3 are envisaged in the short term.

First, some modelling options have not yet been fully evaluated, and will be studied including i) the sampling strategy, ii) adjustment of the number of variables used for building each RF tree, and iii) a further-revised set of predictor variables. Regarding the latter, the inclusion of additional of socio-economic and anthropogenic variables may be important, especially for the monogastric species of chickens, ducks and pigs, for which production is more detached from land constraints and where anthropogenic factors have been shown to be relatively more important [40]. Moreover, the inclusion of different anthropogenic, environmental and land use variables needs to be evaluated species by species.

Second, the benefit of the revised methodology will need to be confirmed for other species and continents and we aim to assess the feasibility and accuracy of producing global runs instead of processing in continental tiles and then merging these to produce global maps.

Third, improvements need to be implemented in i) the automated generation of metadata, ii) the semi-automated updating of inputs (training census data and predictor variables),iii) quality checks on the outputs, especially for anomalous values and iv) the dissemination of outputs for different extents and at different spatial resolutions as required by the users.

## Supporting Information

S1 File Gap-filling evaluation. (DOC)
