# Supplementary material for: Using Random Forest to Improve the Downscaling of Global Livestock Census Data
Source: PLoS One. 2016 Mar 15;11(3):e0150424. doi: 10.1371/journal.pone.0150424 (PMC4792414; doi:10.1371/journal.pone.0150424)
Supplement: S1 File — (DOC) [file pone.0150424.s001.doc]

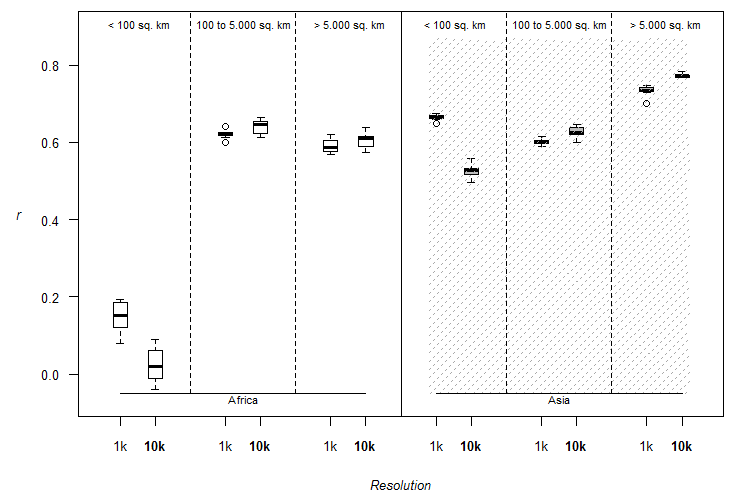


**Figure A. Resolution.** Correlation coefficients between log-transformed observed and predicted densities evaluated through gap-filling, using the stratified regression modelling method, density as dependent variable, the full set of predictor variables, 10 bootstraps, and varying the spatial resolution of the modelling process (1k : 1km or 0.0083333 decimal degrees resolution ; 10k: 10km or 0.083333 decimal degrees resolution). The correlation coefficient is estimated by breaking down evaluation polygons by their size, and for both species and continents.


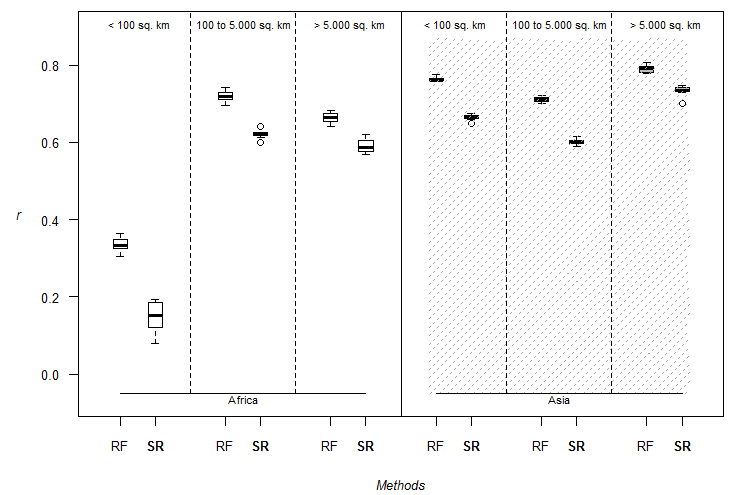


**Figure B. Methods.** Correlation coefficients between log-transformed observed and predicted densities evaluated through gap-filling, using density as dependent variable, the full set of predictor variables, 10 bootstraps, 1 km resolution modelling and varying the modelling method (SR : Stratified regression corresponding to GLW2 ; RF : Random Forest). The correlation coefficient is estimated by breaking down evaluation polygons by their size, and for both species and continents.


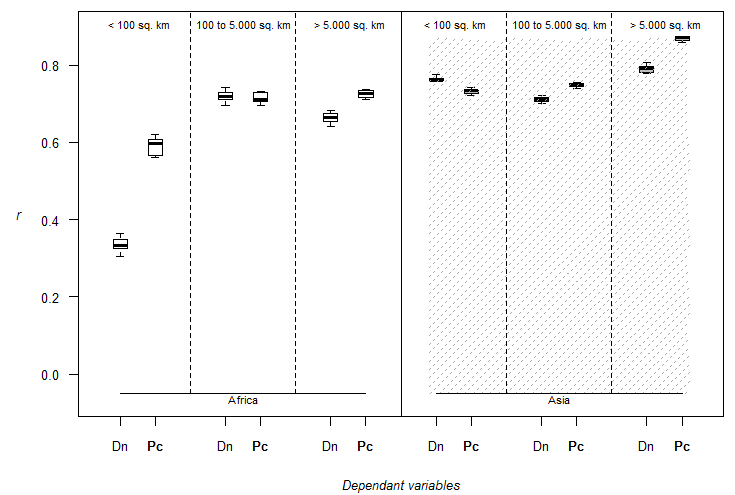


**Figure C. Dependant variable.** Correlation coefficients between log-transformed observed and predicted densities evaluated through gap-filling, using Random Forest as modelling method, the full set of predictor variables, 10 bootstraps, 1 km resolution modelling and varying the dependent variable (Dn : suitability-corrected density corresponding to GLW2 ; Pc : number of animals per capita). The correlation coefficient is estimated by breaking down evaluation polygons by their size, and for both species and continents.


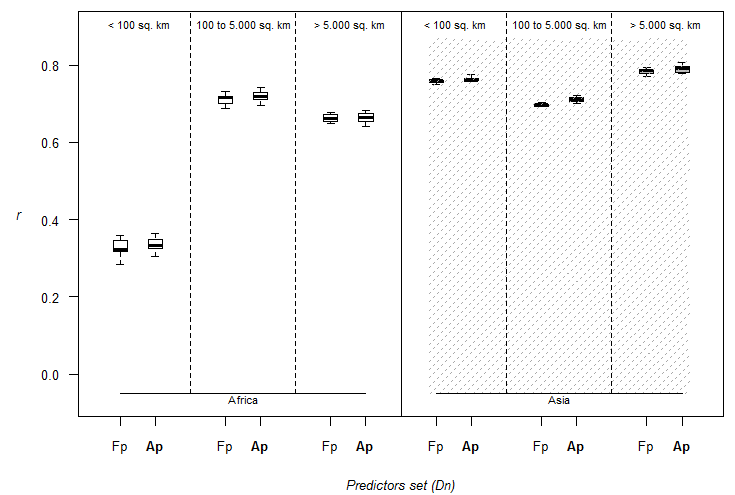


**Figure D. List of Predictors.** Correlation coefficients between log-transformed observed and predicted densities evaluated through gap-filling, using Random Forest as modelling method, density as dependent variable, 10 bootstraps, 1 km resolution modelling and varying the set of predictor variables variable (Ap : all predictors corresponding to GLW2 ; Fp : reduced set of predictor variables). The correlation coefficient is estimated by breaking down evaluation polygons by their size, and for both species and continents.
